# Linking Attention Deficits to Difficulties in the Comprehension of Complex Syntax in Dyslexia

**DOI:** 10.1002/dys.70026

**Published:** 2026-01-29

**Authors:** Mostafa Mazlumi, Mehdi Purmohammad

**Affiliations:** ^1^ Brain and Cognition Clinic Tehran Iran; ^2^ Department of Education University of Alberta Edmonton Canada

**Keywords:** auditory attention, auditory comprehension, dyslexia, passive sentences comprehension, syntax complexity

## Abstract

Recent research suggests that dyslexia involves not only reading difficulties but also deficits in working memory, attention, language, and information processing. This study examined the role of auditory attention in the comprehension of syntactically complex sentences in children with dyslexia. Participants completed an auditory language comprehension test and an auditory sustained attention task. Results showed that the dyslexic group scored lower than controls on both tasks, indicating weaker auditory attention and comprehension. They gave fewer correct answers and exhibited longer reaction times, particularly as syntactic complexity increased. Dyslexic individuals also made more errors and required more time to respond, suggesting impairments in auditory language processing, likely linked to attention deficits. While increased syntactic complexity reduced performance in both groups, the dyslexic group consistently demonstrated more pronounced difficulties than their typical peers did. These findings highlight that auditory attention and syntactic complexity significantly affect sentence comprehension in children with dyslexia. The results suggest that comprehension challenges in dyslexia are not solely due to reading limitations but also involve broader cognitive processing issues. Insights into specific sentence structures and processing delays can inform the development of more sensitive diagnostic tools and targeted interventions for assessing and supporting language comprehension in dyslexic populations.

## 1 Introduction

Developmental dyslexia is a neurobiological learning disorder characterised primarily by difficulties in accurate and/or fluent word recognition, spelling, and decoding skills (Purmohammad 2024). These challenges are often attributed to underlying phonological deficits, which may interact with other cognitive domains such as attention and working memory (Lyon et al. 2003). While dyslexia is typically associated with reading difficulties (Snowling et al. 2020), it is increasingly recognised as a multidimensional disorder that affects a broad range of cognitive and linguistic processes, including syntax and sentence comprehension (Donato et al. 2022). Research has highlighted that children with dyslexia often struggle not only with decoding but also with higher‐order processes such as reading comprehension, particularly when syntactic complexity increases (Stella and Engelhardt 2022). These comprehension difficulties may stem from three primary sources: specific linguistic deficits in syntactic processing, general cognitive limitations that support language understanding, such as working memory (e.g., Wiseheart et al. 2009), and reduced language exposure due to limited reading experience (Robertson and Joanisse 2010).

Attention, particularly auditory attention, has been identified as a key cognitive factor influencing language comprehension in children with dyslexia (Wajuihian and Naidoo 2011). Theories such as the auditory temporal processing hypothesis argue that dyslexic individuals struggle with rapidly changing auditory input, which can impair phonological development and subsequently reading and comprehension abilities (Tallal 1980; Ramus and Szenkovits 2008). Furthermore, sensory‐based models of dyslexia suggest that deficits in selective attention, especially in distinguishing relevant from irrelevant stimuli, may underlie some of the language processing challenges observed in dyslexia (Stein 2023). Despite theoretical and empirical support for the role of attention in language and reading outcomes, there remains a significant gap in understanding how sustained auditory attention specifically affects the comprehension of syntactically complex sentences in dyslexic children. This is particularly important, given that difficulties with passive and non‐canonical sentence structures are well‐documented in both developmental and clinical populations (Stella and Engelhardt 2022).

The present study addresses this gap by examining whether auditory attention performance predicts comprehension of syntactically complex sentences in children with dyslexia. Given the foundational role of attention in auditory language processing (Tallal 1980), this investigation aims to clarify its contribution to sentence‐level comprehension difficulties in dyslexia and to inform more targeted assessment and intervention approaches.

### 1.1 Syntactic Complexity and Sentence Comprehension in Children With Dyslexia

Developmental dyslexia is typically characterised by difficulties in accurate and fluent word recognition, spelling, and decoding abilities. However, recent research suggests that these challenges extend beyond phonological deficits to include broader linguistic impairments, such as syntax and sentence comprehension (Adlof and Hogan 2018), white matter network connectivity deficits (Lou et al. 2019), and even extend to face recognition (Li and Zhao 2025). Understanding how children with dyslexia process syntactically complex sentences is essential, as these structures are common in academic texts and everyday communication.

Passive sentence comprehension has been a central topic of investigation in psycholinguistics, including developmental and clinical contexts such as aphasia (e.g., Caplan and Futter 1986). These structures are of particular interest because they are syntactically more complex than active sentences and deviate from the typical subject–verb–object (SVO) order found in English. In passive constructions, the object appears before the verb and the subject follows it, resulting in a reversal of thematic roles—placing the patient or theme at the beginning and the agent at the end of the sentence. Researchers have typically examined whether listeners can correctly identify the thematic roles in the sentence. Stella and Engelhardt (2022) explored how adults with dyslexia comprehend passive sentences, specifically investigating their reliance on parsing heuristics compared to non‐dyslexic readers. Participants read both active and passive sentences, with semantic plausibility manipulated, while their eye movements were recorded. After each sentence, they answered a comprehension question. The results indicated that dyslexic participants made significantly more comprehension errors, particularly with passive and implausible sentences. Additionally, they exhibited slower reading times overall. These findings suggest that individuals with dyslexia depend more heavily on heuristic strategies during sentence processing than their non‐dyslexic peers.

Studies have shown that children with dyslexia often struggle with syntactic awareness, which refers to the ability to understand and manipulate the grammatical structure of sentences. For instance, Robertson et al. (2024) found that school‐age children with dyslexia performed worse than typically developing peers on syntactic awareness tasks, even after controlling for phonological memory and verbal working memory. However, once phonological awareness was controlled for, group differences were no longer significant, suggesting that phonological deficits may underlie syntactic processing difficulties in dyslexia.

Further supporting the link between syntactic skills and reading comprehension, Kritsotakis and Morfidi (2024) investigated the linguistic abilities of children with specific learning difficulties (SLD), including dyslexia. Their study revealed that morphosyntactic abilities uniquely predicted reading comprehension performance in children with SLD, highlighting the importance of syntactic processing in understanding written texts.

Longitudinal research also indicates that children with language impairments, such as Specific Language Impairment (SLI) or Developmental Language Disorder (DLD), exhibit persistent difficulties in developing clause complexity in their narratives. Araya et al. (2023) observed that while both typically developing children and those with SLI/DLD increased their use of complex syntactic structures over time, the latter group showed ongoing challenges, emphasising the need for targeted interventions to support syntactic development.

Moreover, Georgiou and Theodorou (2023) identified that children with DLD face significant challenges in comprehending complex syntactic constructions. The findings showed that children with DLD have trouble understanding various forms of complex syntax, including object relative clauses, wh‐questions, sentences with non‐standard word order, and passives. Similar difficulties have been observed in English‐speaking children with DLD. These challenges are likely due to difficulties in assigning thematic roles, limitations in working memory, or a combination of both factors. The study suggests that problems with comprehending complex syntax may serve as a universal characteristic of DLD, potentially rooted in grammatical deficits, processing limitations, or both.

A comprehensive meta‐analysis by Georgiou et al. (2022) examined the extent of comprehension deficits in individuals with dyslexia. Analysing data from 76 studies, the review found that individuals with dyslexia exhibited significant deficits in both reading and listening comprehension compared to their typically developing peers. Importantly, the review included studies with both children and adults, revealing that comprehension difficulties are present across age groups, though they may manifest differently depending on task demands and linguistic context. These findings suggest that comprehension difficulties in dyslexia are likely a combination of decoding challenges and broader oral language deficits.

### 1.2 The Role of Attention and Sentence Comprehension in Children With Dyslexia

Attention plays a crucial role in sentence comprehension, particularly when processing syntactically complex structures. Efficient comprehension requires not only linguistic knowledge but also the ability to allocate cognitive resources (see Hubbard and Federmeier 2021), especially sustained and selective attention, to decode and integrate information over time. Moisala et al. (2015), using fMRI, demonstrated that divided and selective attention influences neural activation during sentence comprehension tasks, with increased activity in the left lateral prefrontal cortex—an area implicated in semantic integration and cognitive control. This study was conducted with typically developing adults, indicating how attentional control mechanisms in mature readers support complex sentence processing. These findings also highlight how attentional demands rise when processing complex linguistic input.

Research by Montgomery et al. (2009) further supports this connection, showing that sustained attention and attentional resource allocation significantly correlate with the comprehension of complex sentences in children with language impairments, but not in typically developing peers. This suggests that attention may serve as a limiting factor in language comprehension for children with developmental disorders.

In dyslexia, while traditionally associated with decoding deficits, emerging evidence indicates that auditory attention may also influence language comprehension (Lallier et al. 2019). Specifically, deficits in attentional control may impair the processing of syntactic dependencies, especially in sentences with non‐canonical word order or embedded clauses. Studies have also shown that adults with dyslexia exhibit slower reaction times and reduced accuracy when responding to comprehension questions involving complex syntactic structures, possibly due to competing demands on attention and working memory (Varvara et al. 2014; Hari et al. 2001). These insights collectively suggest that attention deficits may exacerbate comprehension challenges in both children and adults with dyslexia, especially in the presence of syntactic complexity. Accordingly, understanding the attentional mechanisms involved in sentence processing is essential for refining models of language comprehension in developmental disorders.

Despite these insights, there remains a gap in understanding how attentional mechanisms interact with syntactic processing in children with dyslexia. While attention deficits are known to impact language processing, the specific role of attention in comprehending syntactically complex sentences in dyslexic children has not been thoroughly investigated. Addressing this gap is essential for developing comprehensive interventions that target both attentional and syntactic processing skills.

Recent research has emphasised the importance of attentional mechanisms in language comprehension and reading development, particularly in populations with reading difficulties such as dyslexia. Guerra et al. (2024) investigated how auditory attention modulates neural tracking of speech in children with and without dyslexia. Using EEG, they found that dyslexic children exhibited diminished neural tracking of speech signals, especially in noisy auditory environments. Crucially, children with better auditory attention showed stronger neural encoding of speech and better reading skills. These findings suggest a foundational role for auditory attention in language processing, reinforcing the idea that comprehension difficulties in dyslexia may not stem solely from phonological deficits.

Extending this line of inquiry into syntactic processing, Robertson et al. (2024) examined whether children with developmental dyslexia exhibit deficits in syntactic awareness even when phonological processing and memory limitations are controlled. The study revealed that dyslexic children continued to perform significantly worse than their typically developing peers in tasks involving syntactic judgements. This suggests that syntactic processing impairments in dyslexia may constitute an independent deficit rather than a byproduct of other cognitive weaknesses. Given that syntactic comprehension underpins complex sentence understanding, these findings are highly relevant to investigations of language comprehension in dyslexia.

The neurobiological underpinnings of auditory attention deficits in dyslexia were explored by Tschentscher et al. (2018). These researchers utilised diffusion tensor imaging to examine white matter connectivity in adults with dyslexia. They discovered reduced structural connectivity between the left auditory thalamus and the planum temporale—two regions crucial for auditory processing—in individuals with dyslexia. This disconnection could impair the integration of speech sounds necessary for efficient sentence processing and may explain why auditory attention deficits are often observed in this population. Their work provides neural evidence for the attention‐related language processing difficulties commonly reported in dyslexia.

While most studies focus on phonological or auditory pathways, Yeung et al. (2019) offered complementary evidence from the visual domain. They demonstrated that visual attention span (VAS) training improved sentence reading performance in Chinese children with developmental dyslexia. Children who underwent VAS training showed gains in both accuracy and fluency during sentence reading tasks. This finding suggests that attention‐based interventions, whether auditory or visual, can enhance sentence‐level processing, indirectly supporting the idea that attentional capacity modulates syntactic comprehension.

Franceschini et al. (2022) also explored visuo‐spatial attention in children with reading difficulties and found significant impairments in orienting and focusing attention. These deficits correlated strongly with poor reading outcomes, indicating that attentional bottlenecks may be a common thread across multiple forms of language processing difficulty. While the study focused on visual attention, it underscores the broader relevance of attentional control in language comprehension, providing a theoretical bridge to auditory attention processes.

Despite growing evidence supporting the interplay between attention and sentence comprehension in dyslexia and related disorders, findings across populations remain somewhat mixed (e.g., Parrila et al. 2020). Montgomery et al. (2009) examined the role of two dimensions of attention—sustained auditory attention and resource allocation—in children with specific language impairment (SLI) compared to typically developing (TD) peers. Using a continuous auditory performance task and a verbal working memory task, they found that children with SLI performed worse on all attention and sentence comprehension measures. Notably, both attention measures were significantly correlated with complex sentence comprehension in the SLI group, even after controlling for age. In contrast, no such relationship was found in the TD group, suggesting that attentional resources are more critical for language processing in populations with developmental disorders than in typically developing children. While these findings support a link between attention and complex sentence processing in impaired populations, they also indicate that reliance on attention may vary depending on developmental status and linguistic proficiency. This underscores the need to explore how attentional performance, particularly sustained auditory attention, may differentially affect syntactic comprehension in children with dyslexia—a gap our study seeks to address.

Together, these studies highlight a growing consensus that attentional processes—both auditory and visual—are intricately intertwined with the language and reading difficulties encountered in dyslexia. However, a significant gap persists: while much of the literature examined auditory attention or syntactic processing in dyslexia separately, few studies have directly investigated how auditory attention capacity affects the comprehension of syntactically complex auditory sentences. Furthermore, existing evidence is fragmented across age groups, with adult studies primarily addressing neural and cognitive mechanisms (e.g., Tschentscher et al. 2018; Varvara et al. 2014) and child studies focusing on behavioural and attentional outcomes (e.g., Guerra et al. 2024; Yeung et al. 2019). Our study directly addresses this intersection, providing novel insights into how attentional deficits may specifically hinder the real‐time processing of linguistically challenging structures in children with dyslexia.

### 1.3 Rationale and Significance of the Study

Dyslexia is one of the most prevalent learning disorders, making it essential to identify the underlying contributing factors. A better understanding of these factors can inform future research and clinical planning for affected individuals. One such factor is auditory attention, a cognitive function that may influence reading and comprehension abilities in children with dyslexia. While previous research has highlighted the role of auditory attention in specific language impairments and its connection to sentence comprehension, few studies have directly examined the relationship between sustained auditory attention and the comprehension of syntactically complex sentences in individuals with dyslexia. Given the emphasis placed by the DSM‐5 and related studies on attention deficits in the oral comprehension of individuals with dyslexia, further research into this area remains a critical need. Moreover, understanding how attention affects sentence comprehension can inform strategies to support dyslexic learners who may struggle with attentional control during language tasks.

Additionally, although theoretical models predict significant reading comprehension deficits in individuals with dyslexia (Georgiou and Theodorou 2023), empirical findings are mixed. Some studies report notable impairments (e.g., Ferrer et al. 2015), while others find comparable performance to control groups (e.g., Parrila et al. 2020). Some studies examined the role of other cognitive functions, such as working memory, in sentence comprehension. For example, Wiseheart et al. (2009), Stella and Engelhardt (2019), and Sheikhi Karizki and Purmohammad (manuscript in preparation) found that reading comprehension deficits in individuals with dyslexia are mediated by limitations in working memory. Given this inconsistency, it is important to explore potential moderating factors that may explain the variation in outcomes. For this study, we examined the role of attention.

### 1.4 The Present Study

Neurodevelopmental disorders such as dyslexia are often characterised by impairments in a range of cognitive functions, including attention, memory, and processing of sensory stimuli. As a subtype of specific learning disorder, dyslexia is believed to be associated with deficits in auditory attention and syntactic comprehension, which can negatively impact academic learning outcomes. In this study, to conduct a comparative analysis of sentence auditory comprehension in two groups—typically developing students and students with dyslexia—active and passive sentences were used. These sentences were further categorised into plausible and implausible types. Additionally, to examine the correlation between cognitive performance and auditory attention, participants completed an auditory attention test.

Three main research questions were addressed in this study. First, do students with dyslexia differ from typically developing students in their auditory comprehension of sentences? Second, does sentence comprehension vary under different syntactic and semantic conditions? Third, is there a relationship between auditory attention as a cognitive function and auditory sentence comprehension? Then, the overarching goal of this study is to investigate the role of sustained auditory attention in the comprehension of syntactically complex sentences in children with dyslexia and to compare their performance with that of typically developing peers. By analysing behavioural data, we aim to develop a more comprehensive cognitive profile for these individuals, which can support both future research and clinical decision‐making. Ultimately, the findings may help identify more effective intervention strategies.

The study specifically focuses on assessing how auditory attention influences the comprehension of complex syntactic structures in children with dyslexia. Sustained auditory attention is measured as the independent variable using the Sohlberg and Mateer (2001) clinical model, which conceptualises attention as a hierarchical system, with sustained attention forming the base after focused attention. Dependent variables include the accuracy and response time in sentence comprehension tasks. Control variables include age, gender, monolingual language status, nonverbal intelligence, and the absence of neurological disorders. The auditory attention task evaluates participants' ability to maintain focus during repetitive and extended trials, providing a robust measure of sustained auditory attention.

## 2 Method

### 2.1 Participants

Thirty monolingual students aged between 9 and 15 years participated in the study. The sample included two groups: 15 children diagnosed with dyslexia and 15 typically developing controls. Participants with dyslexia were recruited through collaboration with specialised rehabilitation and learning disorder clinics and therapists across Tehran, using email invitations, advertising posters, and direct contact. Typically developing children were recruited through cooperation with schools, principals, and teachers in various districts of Tehran. The overall mean age was 10.7 years (SD = 1.6). The mean age for the dyslexic group was 10.9 years (SD = 1.7), and for the control group, 10.5 years (SD = 1.5). There was no significant age difference between the two groups (*p* > 0.05). Children with dyslexia had been clinically diagnosed by licensed specialists before participation. To control for potential confounds, participants were required to be monolingual, with no history of neurological disorders, and matched on non‐verbal intelligence, age, and gender distribution. Ethical approval for the study was obtained from the Ethics Committee of Shahid Beheshti University (Ethics ID: IR.SBU.REC.1402.118). Informed consent was obtained from all participants and their legal guardians prior to data collection.

### 2.2 Materials and Experimental Design

#### 2.2.1 Auditory Sentence Comprehension Task

The auditory sentence completion task assessed participants' auditory sentence comprehension at the sentence level under four experimental conditions: Active‐Plausible: syntactically simple, semantically plausible, Active‐Implausible: syntactically simple, semantically implausible, Passive‐Plausible: syntactically complex, semantically plausible, and Passive‐Implausible: syntactically complex, semantically implausible. Participants listened to a total of 126 sentences: 6 practice trials, 80 experimental trials (20 items per condition), and 40 grammatically correct filler sentences which were included to reduce response predictability and ensure attention. The 80 target sentences were equally divided between active and passive voice constructions. To control for order effects, all 80 critical items were counterbalanced across two presentation lists, with conditions rotated such that plausible versions of each sentence appeared as implausible in the alternate list, and vice versa. Each sentence contained six words. Within both syntactic categories (active and passive), half of the sentences were semantically plausible (e.g., *The boy ate the apple*), and the other half were implausible (e.g., *The apple ate the boy*).

Each sentence was presented continuously for 4000 (±500) milliseconds. All sentences were recorded by a single speaker (the experimenter) using Standard Persian pronunciation and accent, which also served as one of the inclusion criteria for participant selection. Stimuli were delivered through headphones in a pseudorandomised order for each participant. The total experiment lasted approximately 27 min. To mitigate fatigue, trials were grouped into six blocks, each containing 30 sentences. Comprehension questions followed 40% of the trials and were evenly distributed across blocks.

#### 2.2.2 Auditory Sustained Attention Task

This task measured participants' ability to sustain attention over an extended period in response to repetitive auditory stimuli. The task lasted 15 min and required participants to monitor a stream of auditory word‐pairs, consisting of one animal and one fruit name per pair. Eleven unique stimulus pairs were created, including one designated target (e.g., *apple–horse*) and ten distractor pairs (e.g., *pomegranate–lion*, *grape–horse*). Participants were instructed to press a response key immediately upon detecting the target pair. Stimuli were presented in random order with equal inter‐stimulus intervals. The task was implemented using PsychoPy (Peirce et al. 2019), an open‐source software for designing behavioural experiments.

### 2.3 Procedure

All testing took place in a quiet, well‐lit, and temperature‐controlled laboratory environment. When participants were ready, they initiated the trial by pressing a key. Each sentence was then presented auditorily through headphones and lasted approximately 4000 ms (±500 ms). Three seconds after the sentence ended, a yes/no comprehension question appeared on the screen, targeting a specific grammatical role (e.g., “Did the boy eat the apple?”). Participants had 4000 ms to read the question and 3000 ms to respond using the keyboard. The sentence comprehension task was developed and executed using MATLAB, with custom scripts designed to control stimulus presentation and response timing. Participants sat approximately 1 m from the computer screen and wore headphones throughout the tasks. Instructions were first presented on the screen and explained orally to ensure understanding. Participants used the keyboard to respond.

## 3 Data Analysis

Data was analysed using both parametric and non‐parametric statistical methods, depending on the distribution and nature of the data. Accuracy and reaction times in the attention task were summarised as means and standard deviations. Between‐group comparisons of attention accuracy were conducted using independent‐samples *t*‐tests. Comprehension accuracy and reaction time across different sentence conditions were compared using the Wilcoxon signed‐rank test for within‐group analyses and the Mann–Whitney *U* test for between‐group analyses. Correlations between attention task accuracy and comprehension performance (accuracy and reaction time) were assessed using Spearman's rank correlation coefficient. All statistical tests were two‐tailed, and a significance threshold of *p* < 0.05 was applied.

## 4 Results

The results showed that the mean accuracy percentage in the attention task was 84.39 ± 10.26, and the mean reaction time in the attention task was 639.6 ± 159.2 ms. Additionally, the highest mean number of correct responses to comprehension questions occurred under the active‐plausible condition (2.9 ± 0.9), while the longest mean reaction time in comprehension was observed under the passive‐plausible condition (1.19 ± 0.7 s).

Table 1 presents the percentage accuracy and reaction time in the attention task, as well as the number of correct responses to comprehension questions across different sentence conditions for both the control and dyslexic groups. In the control group, the mean percentage accuracy in the attention task was 93.07 ± 4.75, and the mean reaction time was 655.80 ± 205.72 ms. The highest mean number of correct comprehension responses was observed under the active‐plausible condition (10.8 ± 2.07), while the longest mean reaction time was under the passive‐implausible condition (0.64 ± 0.29 s). In the dyslexic group, the mean percentage accuracy in the attention task was 75.71 ± 5.80, and the mean reaction time was 623.40 ± 98.16 ms. The highest mean number of correct comprehension responses was found in the active‐plausible condition (7.2 ± 2.48), and the longest reaction time occurred under the passive‐plausible condition (1.80 ± 0.36 s). We ran an independent‐samples *t*‐test to estimate whether the difference in attention accuracy (93.08% vs. 75.71%) is statistically significant (*p* < 0.001).

**TABLE 1:** Description of the parameters of the auditory attention and comprehension test in the dyslexic and control groups.

Group	Dyslexia group		Control group	
	Mean	Standard deviation	Mean	Standard deviation
Percentage of correct responses in the attention test	75/7110	5/80562	93/0769	4/75980
Reaction time in the attention test	623/4087	98/16205	655/8013	205/72143
Accuracy in comprehension questions				
Active‐plausible	7/2000	2/48424	10/8000	2/07709
Active‐implausible	3/8000	2/11119	8/4667	2/69568
Passive‐plausible	3/4667	1/76743	8/8667	1/76743
Passive‐implausible	4/6000	1/05560	6/0000	1/51186
Reaction time in comprehension questions				
Active‐plausible	1/5110	0/36873	0/4221	0/33188
Active‐implausible	1/6646	0/49075	0/5787	0/45704
Passive‐plausible	1/8016	0/36067	0/5977	0/32153
Passive‐implausible	1/6877	0/26490	0/6435	0/29552

### 4.1 Comparison of Correct Comprehension Responses Across Sentence Conditions

The Wilcoxon test was used to compare the number of correct comprehension responses across conditions. In the dyslexic group, there were statistically significant differences between most condition pairs, except between the active‐implausible and passive‐plausible conditions (*p* > 0.05). Specifically, in this group, significant differences were observed between the active‐plausible condition and all other conditions, and between the passive‐plausible and passive‐implausible conditions (*p* < 0.05). No significant differences were found between the active‐implausible and passive‐plausible conditions or between the active‐implausible and passive‐implausible conditions (*p* > 0.05). Between‐group comparisons using the Mann–Whitney *U* test showed a significant difference in the number of correct responses across all four sentence conditions (*p* < 0.05), with the control group outperforming the dyslexic group.

### 4.2 Comparison of Reaction Time in Comprehension Tasks Across Conditions

Using the Wilcoxon test, pairwise comparisons of reaction times for comprehension questions in the control group revealed significant differences for all condition pairs except between the active‐implausible and passive‐plausible conditions (*p* > 0.05). In the dyslexic group, significant differences were observed only between active‐plausible vs. passive‐plausible and passive‐plausible vs. passive‐implausible conditions (*p* < 0.05). No significant differences were found among the remaining condition pairs (*p* > 0.05). Between‐group comparisons using the Mann–Whitney *U* test revealed no significant difference in reaction time during the attention task (*p* > 0.05).

### 4.3 Between‐Group Comparison of Comprehension Accuracy

A Mann–Whitney *U* test revealed statistically significant differences between the dyslexic and control groups across all four sentence conditions in terms of comprehension accuracy (*p* < 0.05). The control group consistently had higher accuracy (Table 2).

**TABLE 2:** Comparing accuracy in comprehension questions in different sentences between the dyslexic and control groups.

Group	Mean rank				Sum of ranks				Mann–Whitney *U*				*p*‐Value			
	Active‐plaus	Active‐implau	Passive‐plaus	Passive‐implau	Active‐plaus	Active‐implau	Passive‐plaus	Passive‐implau	Active‐plaus	Active‐implau	Passive‐plaus	Passive‐implau	Active‐plaus	Active‐implau	Passive‐plaus	Passive‐implau
Control	21/43	21/57	22/77	19/53	321/50	323/50	341/50	293/00	23/500	21/500	3/500	52/000	0/000	0/000	0/000	0/010
Dyslexic	9/57	9/43	8/23	11/47	143/50	141/50	123/50	172/00								

### 4.4 Between‐Group Comparison of Reaction Time in Comprehension

A Mann–Whitney *U* test revealed statistically significant differences in reaction time between the dyslexic and control groups across all sentence conditions (*p* < 0.001), with the dyslexic group exhibiting longer reaction times (Table 3).

**TABLE 3:** Comparison of reaction time in answering comprehension questions in different sentences between the dyslexic and control groups.

Group	Mean rank				Sum of ranks				Mann–Whitney *U*				*p*‐Value			
	Active‐plaus	Active‐implau	Passive‐plaus	Passive‐implau	Active‐plaus	Active‐implau	Passive‐plaus	Passive‐implau	Active‐plaus	Active‐implau	Passive‐plaus	Passive‐implau	Active‐plaus	Active‐implau	Passive‐plaus	Passive‐implau
Control	8/20	8/67	8/13	8/18	123/00	130/00	122/00	116/00	3/000	10/000	2/000	1/500	0/000	0/000	0/000	0/000
Dyslexic	22/80	22/33	22/87	22/41	342/00	335/00	343/00	321/00								

### 4.5 Trends in Comprehension Accuracy Across Conditions

**FIGURE 1:**
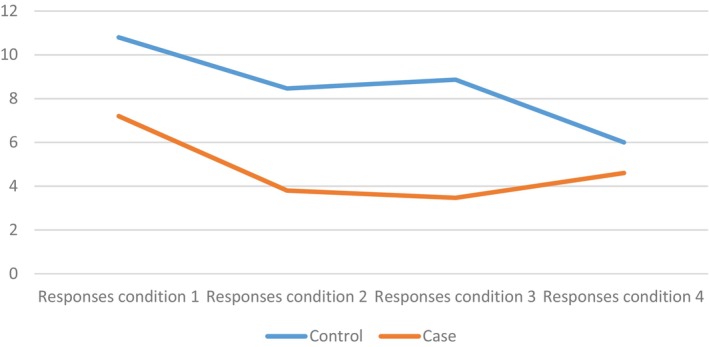
Correct responses across conditions in the two groups.

Figure 1 illustrates changes in comprehension accuracy across sentence conditions in both groups. Increased syntactic and semantic complexity led to a reduction in performance in both groups. Dyslexic participants performed the poorest under the passive‐plausible condition, while controls showed the lowest performance under the passive‐implausible condition.

### 4.6 Trends in Reaction Time Across Conditions

**FIGURE 2:**
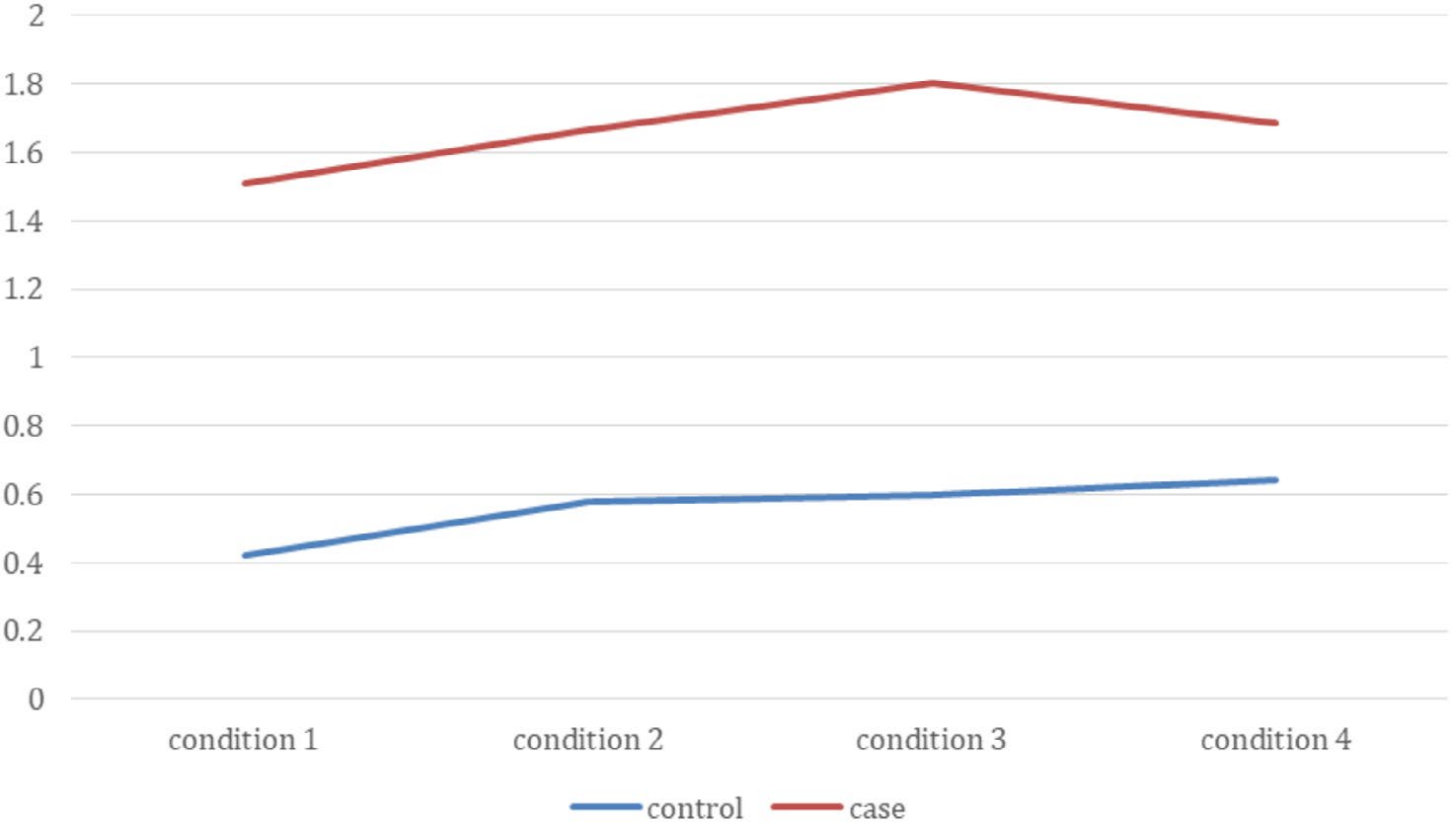
Reaction time for answering comprehension questions in the two groups.

Figure 2 displays changes in reaction time across sentence conditions for both groups. Reaction time was generally longer in the dyslexic group, particularly in conditions with greater syntactic and semantic complexity. The longest reaction time for the control group was in the passive‐implausible condition, whereas for the dyslexic group, it was in the passive‐plausible condition.

### 4.7 Correlation Between Attention Accuracy and Comprehension Accuracy

Spearman correlation analysis was conducted to examine the relationship between attention task accuracy and comprehension accuracy. In the control group, significant correlations were found between attention accuracy and both active‐plausible (*r* = 0.680, *p* < 0.01) and active‐implausible conditions (*r* = 0.547, *p* < 0.05). No significant correlations were observed in the dyslexic group across any condition (*p* > 0.05).

### 4.8 The Relationship Between Attention Test Accuracy and Comprehension Question Performance in the Control Group

To examine the relationship between the percentage of correct responses on the attention test and the number of correct answers to comprehension questions in both the dyslexic and control groups, Spearman's rank correlation was used (Table 4). The results indicated that, in the control group, there was a significant correlation between attention accuracy and comprehension of sentences in the active–plausible and active–implausible conditions (*r* = 0.680, *p* < 0.01 and *r* = 0.547, *p* < 0.05, respectively). However, in the dyslexic group, no significant correlation was found between attention accuracy and performance on any of the four sentence conditions (*p* > 0.05).

**TABLE 4:** Correlation between the percentage of correct answers in the attention test and the number of correct answers in comprehension questions in the Dyslexia group and the control.

Comprehension sentences	Dyslexia group				Control group			
	Active‐plaus	Active‐implau	Passive‐plaus	Passive‐implau	Active‐plaus	Active‐implau	Passive‐plaus	Passive‐implau
Correlation coefficient	0/032	/270	‐0/101	0/092	0/680**	0/547*	0/394	0/452
*p*‐Value	0/909	0/330	0/719	0/744	0/005**	0/035*	0/146	0/091

*Note:* *p < 0.05, **p < 0.01, ***p < 0.001.

### 4.9 The Relationship Between Attention Test Accuracy and Reaction Time in Response to Comprehension Questions in the Control Group

To examine the relationship between the percentage of correct responses on the attention test and reaction time in answering comprehension questions in both the dyslexic and control groups, Spearman's rank correlation was used (Table 5). The results revealed that, in the control group, attention accuracy was significantly negatively correlated with reaction time in the active‐plausible (*r* = −0.631, *p* = 0.012), active‐implausible (*r* = −0.814, *p* < 0.001), passive‐plausible (*r* = −0.527, *p* = 0.044), and passive‐implausible (*r* = −0.645, *p* = 0.009) conditions. However, in the dyslexic group, no significant correlation was found between attention accuracy and comprehension performance in any of the four sentence conditions (*p* > 0.05).

**TABLE 5:** Correlation between the percentage of correct answers in the attention test and the reaction time in response to comprehension questions in the Dyslexia group and the control group.

Comprehension sentences	Dyslexia group				Control group			
	Active‐plaus	Active‐implau	Passive‐plaus	Passive‐implau	Active‐plaus	Active‐implau	Passive‐plaus	Passive‐implau
Correlation coefficient	/004	/186	−/114	−/018	−/631*	−/814**	−/527*	−/645**
*p*‐Value	/990	/508	/685	/950	/012*	/000***	/044*	/009**

*Note:* *p < 0.05, **p < 0.01, ***p < 0.001.

The findings indicate that children with dyslexia showed lower accuracy in the attention task and performed more poorly on sentence comprehension compared to their typically developing peers. Comprehension was particularly challenged when participants processed syntactically and semantically complex sentences, such as passive‐plausible ones, where they exhibited the slowest response times. While both groups were affected by increased complexity, the dyslexic group showed more pronounced difficulties than their typically developing peers. Notably, there was no meaningful relationship between their attention performance and how well they understood or responded to the comprehension questions, unlike the control group, where better attention was linked to both more accurate and faster comprehension. These patterns suggest that the comprehension of syntactically complex sentences in children with dyslexia may not be directly supported by their attentional control in the same way as in typically developing children.

## 5 Discussion

This study examined auditory attention and sentence comprehension of children with and without dyslexia. Participants were equally divided into dyslexic and control groups, matched for mean age. In this study, active and passive sentences were used to conduct a comparative analysis of sentence auditory comprehension in two groups—typically developing children and children with dyslexia. These sentences were further categorised into plausible and implausible types. Additionally, to explore the relationship between cognitive performance and auditory attention, participants completed an auditory attention test. The study addressed three main research questions. First, do students with dyslexia differ from typically developing students in their auditory comprehension of sentences? Second, does sentence comprehension vary under different syntactic and semantic conditions? Third, is there a relationship between auditory attention as a cognitive function and auditory sentence comprehension?

In the auditory sentence comprehension task, the dyslexic group again showed lower accuracy and longer reaction times compared to the control group. These findings suggest that children with dyslexia experience difficulties not only in reading and decoding but also in processing spoken language. This also indicates a general weakness in auditory attentional performance among children with dyslexia. Since all stimuli were presented auditorily rather than in written form, it can be confidently asserted that the comprehension difficulties observed in the dyslexic group are not attributable to decoding deficits. This methodological choice effectively isolates syntactic processing by removing the confounding influence of decoding or grapheme–phoneme conversion difficulties, which are commonly observed in individuals with dyslexia. Therefore, the performance differences between groups likely reflect higher‐level language comprehension processes rather than basic reading impairments. Accuracy was highest for active‐plausible sentences and lowest for passive‐implausible sentences in both groups. Similarly, the fastest reaction times were observed for active‐plausible sentences, while the slowest were found for passive‐plausible sentences, followed closely by passive‐implausible sentences. These patterns also suggest that increasing syntactic and semantic complexity impairs sentence processing, which is reflected in both accuracy and reaction times.

When examining the effects of linguistic complexity, we found that both syntactic and semantic features influenced sentence comprehension. In the control group, accuracy declined progressively from active‐plausible to passive‐implausible sentences, demonstrating that both syntactic passivisation and semantic anomaly independently and jointly contribute to processing difficulty. Interestingly, no significant difference in accuracy was observed between active‐implausible and passive‐plausible sentences in this group, suggesting that the cognitive load imposed by syntactic or semantic complexity alone might be comparable. These findings align with Stella and Engelhardt (2022) and Wiseheart et al. (2009), highlighting that dyslexia involves broader deficits in sentence‐level language comprehension, especially under increased processing demands.

In the dyslexic group, a similar pattern was observed, but with notable differences. Not only was their overall performance poorer, but they also showed no significant difference between active‐implausible and passive‐implausible sentences. This may indicate that syntactic complexity poses a greater challenge for these children than semantic complexity alone. The finding aligns with prior research showing that individuals with dyslexia often struggle more with syntactically complex constructions than with semantically anomalous content. In other words, while both dimensions of linguistic complexity affect sentence comprehension, syntactic factors may have a more profound impact on dyslexic learners. These results corroborate findings from Stella and Engelhardt (2022), who examined sentence comprehension among university students with and without dyslexia using a visual task. Similarly, they found that comprehension of syntactically and semantically complex sentences was significantly impaired in the dyslexic group. Despite differences in participant age and modality (visual vs. auditory), both studies suggest that dyslexia entails broader deficits in sentence‐level language comprehension, particularly under increased processing demands.

Our findings also align with those of Wiseheart et al. (2009), who demonstrated that young adults with developmental dyslexia showed poorer performance than controls in interpreting passive and syntactically embedded sentences. Dyslexic participants in that study exhibited reduced accuracy and increased reaction times when processing syntactically complex structures, particularly passives, which resonates with our results showing prolonged processing times and lower comprehension accuracy in dyslexic children for passive constructions.

The reaction time findings in our study further underscore the role of linguistic complexity in comprehension. In the control group, reaction times increased steadily from active‐plausible to passive‐plausible sentences, reflecting the incremental cognitive load associated with processing increasingly complex sentence types. Although passive‐implausible sentences showed slightly faster reaction times than passive‐plausible ones, this might be due to participants recognising the semantic anomaly and responding more quickly despite poor accuracy. In the dyslexic group, the longest reaction times were observed for passive‐plausible sentences, suggesting that syntactic complexity alone may be especially taxing. Interestingly, reaction times decreased again for passive‐implausible sentences, possibly due to early detection of semantic incongruity, leading to premature or superficial responses. These findings imply that children with dyslexia may engage in more prolonged processing of syntactically demanding inputs, especially when these inputs retain some degree of semantic plausibility.

Furthermore, the observed patterns suggest that auditory attention and sentence comprehension are likely interrelated cognitive functions. The dyslexic group's lower accuracy in the auditory attention task may partly account for their poorer performance in sentence comprehension, particularly under conditions requiring sustained attention to morphosyntactic structure. This interpretation is supported by the fact that the dyslexic group did not differ significantly from the control group in reaction time during the attention task, reinforcing the notion that accuracy (rather than speed) is the more sensitive marker of attentional deficits in this population. These results also support theoretical models proposing that dyslexia involves domain‐general attentional deficits, particularly in the auditory modality (Pennington 2006; Astle et al. 2009; Facoetti et al. 2010; Ruffino et al. 2010; Krause 2015). Sustained attention appears to contribute to the effective parsing, integration, and interpretation of complex sentences.

In light of these findings, it appears that dyslexia is not limited to deficits in visual decoding or phonological processing. Instead, it also involves difficulties in real‐time comprehension of spoken sentences, especially when processing syntactic structures that deviate from canonical word order. Our study adds to a growing body of evidence highlighting sentence‐level comprehension deficits in dyslexia and suggests that both syntactic and semantic complexity significantly modulate these difficulties. Supporting this interpretation, Stella and Engelhardt (2019) found that participants with dyslexia were poor in the comprehension of syntactic ambiguities. Their results identified that group (or dyslexia status) was significantly correlated with comprehension in the ambiguous‐optional and unambiguous‐reflexive conditions. Also, in our study, dyslexic students performed relatively poorly on syntactically complex sentences despite having similar attentional reaction times, which may point to limitations in syntactic processing that are not fully explained by attentional capacity alone.

In summary, the findings suggest that syntactic complexity imposes a significant burden on sentence comprehension for all children, but disproportionately so for those with dyslexia. While semantic anomaly also disrupts comprehension, its effects may be secondary to those of syntactic structure—particularly in dyslexic individuals. Furthermore, the parallel deficits observed in both auditory attention and sentence comprehension raise the possibility of shared cognitive mechanisms. These insights underscore the need for interventions that address both syntactic processing and attentional control to support the language development of children with dyslexia.

### 5.1 The Role of Auditory Attention in Sentence Comprehension

One of the central aims of this study was to investigate the potential relationship between auditory attention, as a cognitive function, and auditory sentence comprehension in children with and without dyslexia. Our findings provide critical insight into this issue. The results of the sustained auditory attention task revealed that children with dyslexia demonstrated significantly lower accuracy (*M* = 75.71%, SD = 5.80) compared to their typically developing peers (*M* = 93.07%, SD = 4.75). Interestingly, no significant difference was observed between the two groups in terms of reaction time (dyslexic group: *M* = 623.40 ms; control group: *M* = 655.80 ms). Given that in sustained attention paradigms, accuracy is generally considered a more sensitive and meaningful indicator of attentional performance than reaction time (Parasuraman 1998), these findings suggest that children with dyslexia may indeed exhibit deficits in auditory attentional control.

Moreover, these attentional deficits appear to parallel the group differences observed in sentence comprehension performance. The dyslexic group consistently showed fewer correct responses to comprehension questions across all sentence conditions, especially under more syntactically complex (passive) or semantically implausible conditions. Notably, their performance was highest in the active‐plausible condition and lowest in the passive‐implausible condition—a pattern that suggests greater sensitivity to both syntactic complexity and semantic incongruity.

The convergence of poorer auditory attention and lower comprehension accuracy in the dyslexic group raises the possibility of a functional relationship between these two cognitive domains. Auditory sentence comprehension, particularly under real‐time processing demands, relies on the ability to sustain attention, inhibit irrelevant stimuli, and maintain task‐relevant information in working memory (Stevens et al. 2006). Deficits in auditory attention may, therefore, constrain a dyslexic child's ability to effectively parse, integrate, and interpret spoken sentences, especially when linguistic input is complex or unexpected.

These findings are consistent with prior studies that have reported reduced performance on attention tasks in individuals with dyslexia (Facoetti et al. 2010; Ruffino et al. 2010). Specifically, Facoetti et al. (2010) have shown that dyslexic individuals demonstrate difficulties in both visual and auditory attention, which in turn may negatively impact phonological and lexical processing. More recent research also supports a broader view of dyslexia as involving domain‐general attentional deficits, particularly in the auditory modality (Astle et al. 2009; Krause 2015). The present study adds to this literature by linking auditory attention performance directly with real‐time auditory sentence comprehension.

In contrast to the control group, which showed relatively fast and accurate responses even under syntactic and semantic manipulation, the dyslexic group required more time and produced more errors, especially under passive‐implausible conditions. This suggests that attentional limitations may be especially disruptive in situations that place high demands on both syntactic parsing and semantic integration.

In summary, the current study highlights that syntactic complexity disproportionately affects children with dyslexia and that auditory attention plays a key role in real‐time sentence comprehension. These findings contribute to the limited literature on auditory sentence comprehension in dyslexia and suggest directions for future research and practical interventions. Taken together, these findings underscore the importance of considering attentional processes—particularly auditory attention—as a potential source of comprehension difficulty in dyslexia. They also support theoretical models that frame dyslexia not merely as a phonological disorder but as a multifactorial condition involving deficits in broader cognitive systems, including attention (Pennington 2006).

## 6 Limitations and Future Directions

Several limitations should be noted. First, the sample size was relatively small, which may limit generalizability. Second, the study focused on a single modality (auditory), and future research could benefit from cross‐modal comparisons. Third, additional neurocognitive measures could provide more fine‐grained insight into the mechanisms linking attention and sentence comprehension. Future studies with larger samples and more fine‐grained neurocognitive measures are warranted to further disentangle the contributions of auditory attention to sentence processing in dyslexia. Additionally, the design of interventions targeting attentional control may represent a promising avenue for improving comprehension outcomes in children with dyslexia.

Children with dyslexia often exhibit attention difficulties, which can make it challenging for them to follow verbal instructions, particularly in noisy or complex listening situations (Guerra et al. 2024). Implementing noise‐reduction strategies in classrooms and other learning environments may help support their learning.

Moreover, future studies that better clarify which types of attention are most important for learning to read could help improve diagnostic tools. As a result, they could help determine whether some children would benefit from attention training alongside traditional reading interventions, like phonological or letter‐sound instruction.

## Funding

The authors have nothing to report.

## Conflicts of Interest

The authors declare no conflicts of interest.

## Data Availability

The data that support the findings of this study are available from the corresponding author upon reasonable request.
